# Supplementary material for: Polygenic Score for Body Mass Index Is Associated with Disordered Eating in a General Population Cohort
Source: J Clin Med. 2020 Apr 21;9(4):1187. doi: 10.3390/jcm9041187 (PMC7231239; doi:10.3390/jcm9041187)
Supplement: Supplementary file 1 [file jcm-09-01187-s001.pdf]

Quality control checks of genotyping data

Individuals with disproportionate levels of individual missingness (i.e., >3%), insufficient sample replication (identity by descent < 0.1), biological sex mismatch, and non-European ancestry (as defined by multi-dimensional scaling using the HapMap Phase II, release 22, reference populations) were excluded. SNPs with a minor allele frequency (MAF) of < 1%, excessive missingness (i.e., call rate < 95%), or a departure from the Hardy–Weinberg equilibrium (*P* value $< 5 \times 10^{-7}$) were removed. Imputation was conducted with Impute3 using the HRC 1.0 as the reference panel [1] and phasing was carried out using ShapeIT (v2.r644). Finally, post-imputation quality control checks were performed; any SNPs with MAF less than 1%, Impute3 information quality metric of < 0.8, and not confirming to Hardy-Weinberg equilibrium ($P < 5 \times 10^{-7}$) were removed. After data cleaning, a total of 8,654 individuals (4,225 females and 4,429 males) and 4,054,653 SNPs remained eligible for analyses.

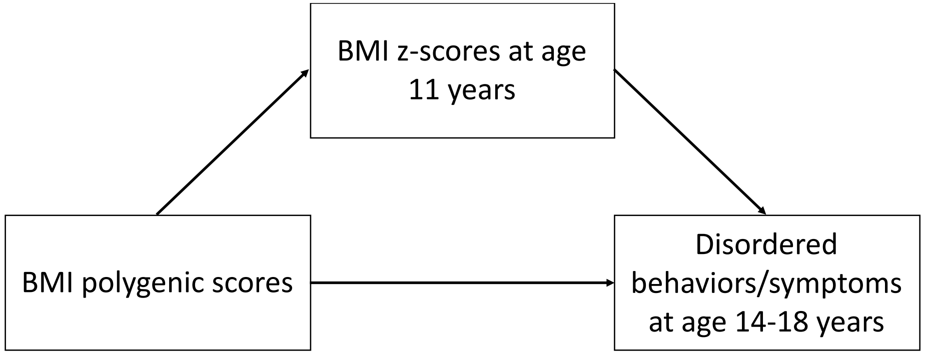

Figure 1. Directed acyclic graph (DAG) of the causal mediation analyses. The body mass index polygenic scores (BMI-PGS) was derived from summary statistics of the genome-wide association study (GWAS) carried out by the Genetic Investigation of Anthropometric Traits (GIANT) consortium [8] and were calculated for participants in the Avon Longitudinal Study of Parents and Children [4–7]. The sex and age adjusted BMI z-scores were included as a mediator in the causal mediation analyses that was carried out using the R package ′mediation′ (version 4.4.6; 32). The 'mediation' package is based on concepts proposed in modern causal inference [9]. Prior to the mediation analyses the BMI-PGS was standardized (to mean zero and standard deviation of one) and the analyses were controlled for biological sex and the first four ancestry-informative principal components. Confounders are not included in this DAG for simplicity.

Table 1. Age, sex, and ethnicity of the participants in the Avon Longitudinal Study of Parents and Children at all three time points.

Wave	Age in years (SD)	Female N (%)	Child ancestry/ white ethnicity N (%)
14	14.0 (0.19)	3,416 (55.5)	5,372 (87.2)
16	16.7 (0.24)	3,095 (58.7)	4,482 (86.0)
18	18.7 (0.49)	2,174 (64.2)	3,010 (88.9)

Table 2. Associations of body mass index polygenic score (BMI-PGS) with BMI in the Avon Longitudinal Study of Parents and Children.

Outcome	Age outcome measured (years)	N	Threshold[a]	N SNPs	R^2	β (95% CI)[b]	Q[c]
BMI	11	5371	0.0180001	19457	0.110480	1.14 (1.05, 1.22)	9.09×10^{-5}
BMI z-scores	11	4037	0.0180001	19457	0.123235	0.40 (1.05, 1.22)	9.09×10^{-5}
BMI	18	3931	0.0230001	21040	0.121126	1.44 (1.31, 1.56)	9.09×10^{-5}

SNP = single nucleotide polymorphism; R^2, squared multiple correlation; OR, odds ratio; CI, confidence interval. [a] The optimal *P*-value threshold for the inclusion of SNPs in the calculation of the body mass index (BMI) polygenic score (PGS) as determined by PRSice's high-resolution scoring [2]. [b] Standardized betas reflect one standard deviation increase in the standardized (to mean zero and standard deviation of one) BMI-PGS. [c] Benjamini & Hochberg false discovery rate adjusting for the number of phenotypes tested [3].

Table 3. Effect size of associations of body mass index polygenic score (BMI-PGS) with disordered eating behaviors and cognitions in the total samples and separately for female participants of the Avon Longitudinal Study of Parents and Children.

		Total sample	Females[a]
**Binary outcomes**	**Age outcome measured (years)**	**OR (95% CI)[b]**	**OR (95% CI)[b]**
Fasting	14	1.45 (1.28, 1.64)	1.43 (1.25, 1.64)
Fasting	16	1.30 (1.18, 1.43)	1.27 (1.15, 1.42)
Fasting	18	1.26 (1.06, 1.51)	1.26 (1.05, 1.52)
Binge eating	14	1.29 (1.14, 1.47)	1.27 (1.08, 1.49)
Binge eating	16	1.22 (1.10, 1.35)	1.18 (1.05, 1.33)
Binge eating	18	1.23 (1.10, 1.39)	1.25 (1.09, 1.43)
Purging	16	1.24 (1.08, 1.42)	1.23 (1.07, 1.42)
Purging	18	1.21 (1.03, 1.43)	1.21 (1.02, 1.44)
**Continuous outcomes**	**Age outcome measured (years)**	**β (95% CI)[c]**	**β (95% CI)[c]**
Thin ideal internalization	14	-0.15 (-0.23, -0.07)	-0.15 (-0.24, 0.05)
Body dissatisfaction	14	0.99 (0.77, 1.22)	1.24 (0.92, 1.55)
Restrained eating	14	0.14 (0.10, 0.17)	0.16 (0.11, 0.21)
Emotional eating	14	0.21 (0.052, 0.38)	-0.10 (-0.34, 0.14)
External eating	14	-0.19 (-0.30, -0.09)	-0.18 (-0.32, -0.05)

OR, odds ratio; CI, confidence interval. [a] Polygenic score analyses only carried out in female participants. [b] ORs reflect one standard deviation change in the standardized (to mean zero and standard deviation of one) BMI-PGS. [c] Standardized betas reflect one standard deviation increase in the standardized (to mean zero and standard deviation of one) BMI-PGS.

Table 4. Generalized linear mixed models for the association between the body mass index polygenic score (BMI-PGS) and the disordered eating behaviors with age at self-report of the disordered eating as an interaction term [a].

Outcome	Threshold [b]	N SNPs	χ^2	$P_{interaction}$
Fasting	1	81,503	4.89	0.09
Binge eating	1	81,503	5.23	0.07
Purging	1	81,503	0.19	0.66

SNP = single nucleotide polymorphism; χ^2, test statistic as measured by chi-square. [a] Analyses were corrected for biological sex and the first four ancestry-informative principal components for the individuals in the Avon Longitudinal Study of Parents and Children. [4–7]. [b] *P*-value threshold for the inclusion of SNPs in the calculation of the BMI-PGS was set to 1 using PRSice [2].

References

1. McCarthy, S.; Das, S.; Kretzschmar, W.; Delaneau, O.; Wood, A.R.; Teumer, A.; Kang, H.M.; Fuchsberger, C.; Danecek, P.; Sharp, K.; et al. A reference panel of 64,976 haplotypes for genotype imputation. *Nat. Genet.* **2016**, *48*, 1279–1283.
2. Euesden, J.; Lewis, C.M.; O'Reilly, P.F. PRSice: Polygenic Risk Score software. *Bioinformatics* **2015**, *31*, 1466–1468.
3. Benjamini, Y.; Hochberg, Y. Controlling the False Discovery Rate : a Practical and Powerful Approach to Multiple Testing. *J. R. Stat. Soc. Ser. B* **1995**, *57*, 289–300.
4. Boyd, A.; Golding, J.; Macleod, J.; Lawlor, D.A.; Fraser, A.; Henderson, J.; Molloy, L.; Ness, A.; Ring, S.; Smith, G.D. Cohort profile: The 'Children of the 90s'-The index offspring of the avon longitudinal study of parents and children. *Int. J. Epidemiol.* **2013**, *42*, 111–127.
5. Fraser, A.; Macdonald-wallis, C.; Tilling, K.; Boyd, A.; Golding, J.; Davey smith, G.; Henderson, J.; Macleod, J.; Molloy, L.; Ness, A.; et al. Cohort profile: The avon longitudinal study of parents and children: ALSPAC mothers cohort. *Int. J. Epidemiol.* **2013**, *42*, 97–110.
6. Golding; Pembrey; Jones; The Alspac Study Team ALSPAC-The Avon Longitudinal Study of Parents and Children. *Paediatr. Perinat. Epidemiol.* **2001**, *15*, 74–87.
7. Golding, J.; Team, S. The Avon Longitudinal Study of Parents and Children (ALSPAC) – study design and collaborative opportunities. **2004**, 119–123.
8. Yengo, L.; Sidorenko, J.; Kemper, K.E.; Zheng, Z.; Wood, A.R.; Weedon, M.N.; Frayling, T.M.; Hirschhorn, J.; Yang, J.; Visscher, P.M. Meta-analysis of genome-wide association studies for height and body mass index in ~700000 individuals of European ancestry. *Hum. Mol. Genet.* **2018**, *27*, 3641–3649.
9. VanderWeele, T. *Explanation in causal inference: methods for mediation and interaction*; Oxford University Press, 2015;
